# Synergic effect of Eicosapentaenoic acid and Lovastatin on gene expression of HMGCoA reductase and LDL receptor in cultured HepG2 cells

**DOI:** 10.1186/1476-511X-9-135

**Published:** 2010-11-30

**Authors:** Maria Notarnicola, Caterina Messa, Maria G Refolo, Valeria Tutino, Angelica Miccolis, Maria G Caruso

**Affiliations:** 1 Laboratory of Biochemistry, National Institute for Digestive Diseases Castellana Grotte, Bari, Italy

## Abstract

**Background:**

PUFAs are potent inhibitors of 3-hydroxy-3-methylglutaryl coenzyme A (HMG-CoA) reductase, an enzyme catalyzing the conversion of HMGCoA to mevalonate, the rate limiting step in cholesterol biosynthesis. Statins represent a class of drugs that are widely used to treat hypercholesterolemia for their ability to inhibit cholesterol biosynthesis and to up-regulate the synthesis of Low Density Lipoprotein (LDL) receptors in the liver. PUFAs mediate many, if not all, actions of statins and this could be one mechanism by which they lower cholesterol levels. The purpose of this study was to investigate whether combined treatment with Eicosapentaenoic acid (EPA) and lovastatin enhanced the regulatory effect on gene expression of HMGCoA reductase and LDL receptor in HepG2 cell line.

**Results:**

The combined treatment with EPA and lovastatin enhanced the regulatory effect on gene expression of HMGCoA reductase and LDL receptor in HepG2 cell line. Moreover, we detected a synergistic effect on the inhibition of cancer cell proliferation obtained by combination of EPA and Lovastatin.

**Conclusions:**

The use of EPA, in combination with low doses of Lovastatin may have potential value in treatment of neoplastic diseases.

## Introduction

Long-chain polyunsaturated fatty acids (PUFAs), named for the position of their terminal double bond, the n-6 and n-3 long-chain PUFAs, are part of the phospholipid structure of all membranes and play additional roles as signaling molecules and modulators of gene expression [1-3]. Long-chain PUFAs may be directly consumed in the diet or synthesized from their essential fatty acid precursors, linoleic acid (LA) and α-linolenic acid (LNA) [4].

Clinical studies from cardiovascular medicine, psychiatry and other disciplines have demonstrated correlations between low n-3 PUFA levels and increased disease risk [5,6] and have shown that increasing n-3 levels by diet or supplementation may confer a variety of health benefits [1,7,8]. A major effect of n-3 PUFA is to lower plasma triacylglycerols and lipoprotrein concentrations, in normal as well as hypertriglyceridaemic subjects [9].

PUFAs are potent inhibitors of 3-hydroxy-3-methylglutaryl coenzyme A (HMG-CoA) reductase, an enzyme catalyzing the conversion of HMGCoA to mevalonate, the rate limiting step in cholesterol biosynthesis. PUFAs mediate many, if not all, actions of statins [10] and this could be one mechanism by which they lower cholesterol levels.

Statins represent a class of drugs that are widely used to treat hypercholesterolemia for their ability to inhibit cholesterol biosynthesis and to up-regulate the synthesis of Low Density Lipoprotein (LDL) receptors in the liver [11].

Statins having biochemical effects on cholesterol synthesis, are considered as potential anti-tumor agents [12], inhibiting tumor cell growth by restricting either cholesterol availability or cholesterol synthesis [12,13]. Nevertheless, the use of statins in cancer trials has been greatly limited by their high-dose toxicity that is characterized by severe myopaty [14].

An important feature of malignant transformation is the loss of the cholesterol feedback inhibition mechanism that regulates cholesterol synthesis. The main cholesterol feedback defect in malignant cells has been located at the HMGCoA reductase step.

Cancer cells seem to require an increased concentration of cholesterol and cholesterol precursors and this requirement may be fulfilled by increased HMGCoA reductase activity.

In our previous study, HMGCoA reductase activity was found to be enhanced in human colorectal cancer that did not express LDL receptors [15], indicating that LDL receptors absence, which deprives colonic neoplastic cells of exogenous sterols, is overcome by an increase in endogenous cholesterol synthesis.

Several studies [15-17] have demonstrated that the absence of LDL receptor induces neoplastic cells to depend on endogenous cholesterol synthesis for their proliferation, resulting in an increase of HMGCoA reductase activity inside the cells. Several studies showed that HMGCoA inhibitor blockade of mevalonate synthesis induced cell cycle arrest *in vitro *[18-20] and inhibited tumor growth in vivo [21].

Previously, we have demonstrated an anti-proliferative effect of simvastatin in two human colon cancer cell lines [16]. Simvastatin inhibited cell proliferation at pharmacological doses in DLD-1 and Caco2 cell lines. The growth inhibition by simvastatin observed in DLD-1 cells was mediated by a proapoptotic effect, whereas in Caco2 cells the anti-proliferative effect takes place regardless of apoptosis.

In the present study we investigated *in vitro *whether combined treatment with Eicosapentaenoic acid (EPA), belonging to omega-3 family, and lovastatin enhanced the regulatory effect on gene expression of HMGCoA reductase and LDL receptor in HepG2 cell line.

## Materials and methods

### Cell culture conditions

HepG2, a cell line derived from human liver tissue with a well differentiated hepatocellular carcinoma, were obtained from the ICLC (IST, Genoa, Italy).

Cells were routinely cultured in DMEM (Dulbecco's modified Eagle's medium) supplemented with 10% FBS (fetal bovine serum), 100 U/ml penicillin, 100 μg/ml streptomycin, in monolayer culture, and incubated at 37°C in a humidified atmosphere containing 5% CO_2 _in air. At confluence, the grown cells were harvested by means of trypsinization and serially subcultured with a 1:4 split ratio. All cell culture components were purchased from Sigma-Aldrich (Milan, Italy).

### Lovastatin and EPA treatment

To elucidate the effect of Lovastatin and EPA on HMG-CoA reductase, LDL receptor gene expression and cell proliferation, HepG2 cells were plated at a density of 3 × 10^5 ^cells/5 ml of DMEM containing 10% FBS in 60-mm tissue culture dishes (Corning Costar Co., USA).

Separate plates were seeded for each assay and when the cells were approximately 60% confluent were exposed to the treatment.

To examine the response to EPA, HepG2 cells were treated for 48 h with culture medium supplemented with various concentrations of EPA (1, 10, 25, 50 and 100 μM). Each experiment included a control without EPA and a control with the same amount of DMEM -BSA used for dissolving the fatty acid.

To evaluate Lovastatin effect on HepG2 cells, the drug dissolved in 0,1 N NaOH was added to the culture medium at increasing concentrations (1, 10, 25, 50 and 100 μM) and incubated for 48 hours. Each experiment included an untreated control and a control that received the same amount of NaOH used for adding Lovastatin.

For the experiment testing the effect of combined treatment of Lovastatin and EPA on subconfluent HepG2 cells two sets of experiments were prepared. In the first set, HepG2 cells were incubated with 50 μM of EPA and increasing concentrations of Lovastatin (1,10, 25, 50 and 100 μM). The second set of experiments were performed by incubating HepG2 with Lovastatin at the concentration of 50 μM with increasing concentrations of EPA (1,10, 25, 50 and 100 μM) (data not shown). In these experimental conditions, HepG2 cells were allowed to growth for 48 h. Each experiment included an untreated control and a control with the equivalent concentration of solvent used for adding Lovastatin and EPA.

Triplicate culture were set up for each compound concentration and for control, and each experiment was repeated 4 times. Cell viability, determined using the trypan blue exclusion test, always exceeded 90%.

### LDL receptor and HMG-CoA reductase gene expression

Analysis of gene expression was performed in HepG2 cells treated with 1, 10, 25, 50 and 100 μM of EPA and Lovastatin alone and with both compounds at established concentrations for 48 hours.

Cells were washed twice in phosphate buffered saline (PBS) and then trypsinized and centrifuged at low speed. The cell pellets were resuspended in 0.3 ml pure distilled water and used for RNA extraction.

Total cell RNA was isolated with TRI-Reagent (Mol. Res. Centre Inc. Cincinnati, USA), following the manufacturer's instruction. Total cell RNA was extracted using Tri-Reagent (Mol. Res. Center Inc., Cincinnati, Ohio, USA), following the manifacture's instruction. About 2 μg total cell RNA, extracted from both the control and treated cells, was used for cDNA synthesis. Reverse transcription (RT) was carried out in 20 μl of the final volume at 41°C for 60 min, using 30 pmol antisense primer (Table 1) for analyses of the HMGCoA reductase, LDL receptor and β-actin gene. The β-actin gene was utilized as an internal control and was chosen as a reference gene because it is a housekeeping gene.

**Table 1:** Sequences of amplification primers

Gene		Primer
LDL receptor	Sense	5'-CAATGTCTCACCAAGCTCTG-3'
	Antisense	5'-TCTGTCTCGAGGGGTAGCTG-3'
HMG-CoA reductase	Sense	5'-TACCATGTCAGGGGTACGTC-3'
	Antisense	5'-CAAGCCTAGAGACATAATCATC
β-actin	Sense	5'-AAAGACCTGTACGCCAACACAGTGCTGTCTGG-3'
	Antisense	5'-CGTCATACTCCTGCTTGCT GATCCACATCTGC-3'

Real-time PCRs were performed in 25 μl final volume containing 2 μl cDNA, master mix with SYBR Green (iQ SYBR Green Supermix; Bio-Rad, Milan, Italy) and sense and antisense primers for HMGCoA reductase, LDL receptor and β-actin gene (Table 1).

Real-Time PCR was carried out with iCycler Thermal Cycler System apparatus (Bio-Rad) using the following parameters: one cycle of 95°C for 1 min and 30 s, followed by 45 cycles at 94°C for 10 s, 55°C for 10 s and 72°C for 30 s and a further melting curve step at 55-95°C with a heating rate of 0.5°C per cycle for 80 cycles. The PCR products were quantified by external calibration curves, one for each tested gene, obtained with serial dilution of known copy number of molecules (10^2^-10^7 ^molecules). All expression data were normalized by dividing the amount of target by the amount of β-actin used as internal control for each sample. The specificity of the PCR product of each tested gene was confirmed by gel electrophoresis.

### Assessment of cell proliferation

After Lovastatin and/or EPA treatment for 48 hours, the proliferative response was estimated by colorimetric 3-(4,5 di-methylthiazol-2-yl)-2,5-diphenyltetrazolium bromide (MTT) test. In brief, MTT stock (5 mg/ml in medium) was added to each dish at a volume of one-tenth the original culture volume and incubated for 2 hours at 37°C in humidified CO_2_. At the end of the incubation period, the medium was removed, and blue formazan crystal were solubilized with acidic isopropanol (0.1 N HCl in absolute isopropanol). MTT conversion to formazan by metabolically viable cells was monitored by spectrophotometer at an optical density of 570 nm.

### Statistical analysis

The significance of the differences between the control group and each experimental group was evaluated with one way analysis of variance and the Dunnett' post test. Differences were considered significant at a 5% probability level.

## Results

**Figure 1:**
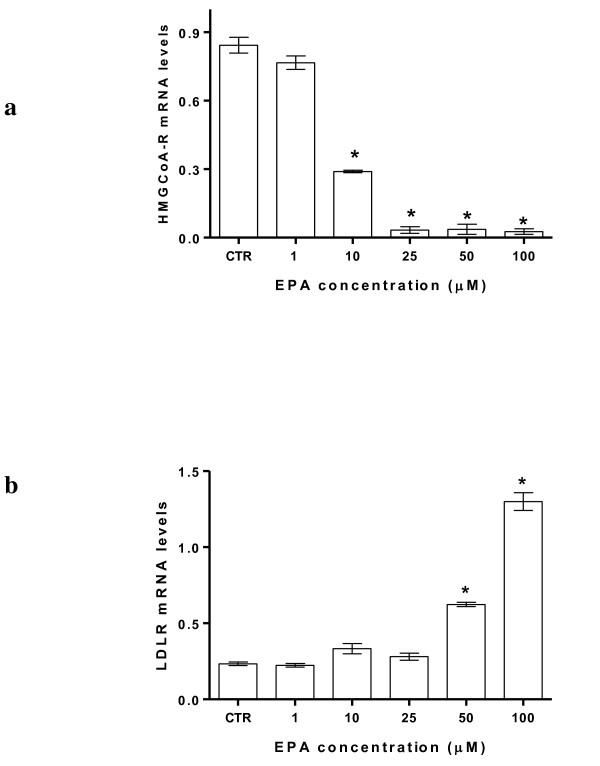
**EPA effects on HMGCoA reductase (panel a) and LDLR (panel b) mRNA levels in HepG2 cells**. All data represent the mean ± SE of four consecutive experiments. mRNA levels are expressed as ratio of the amount of gene target by the amount of β-actin. *P *value was determined by one way analysis with Dunnett' post test. *P < 0.05 versus control

Results showed that EPA significantly inhibited HMGCoA reductase gene expression and up-regulated mRNA LDL receptor (Figure 1a and 1b).

**Figure 2:**
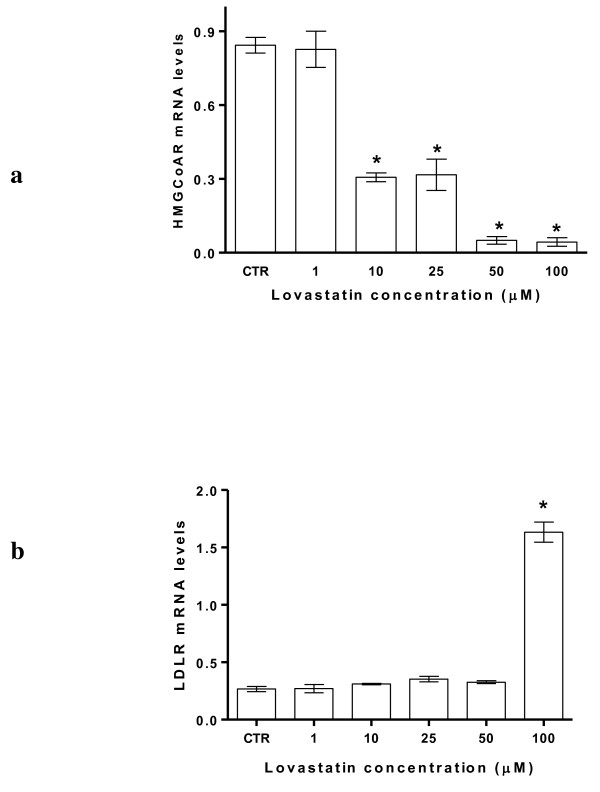
**Lovastatin effect on HMGCoA reductase (panel a) and LDLR (panel b) mRNA levels in HepG2 cells**. All data represent the mean ± SE of four consecutive experiments. mRNA levels are expressed as ratio of the amount of gene target by the amount of β-actin. *P *value was determined by one way analysis with Dunnett' post test. *P < 0.05 versus control

The treatment of HepG2 cells with increasing concentrations of Lovastatin caused a similar effect to that induced by EPA (Figure 2a and 2b). The regulatory effect on LDLR receptor gene expression was significantly evident at higher concentrations (Figure 2b).

**Figure 3:**
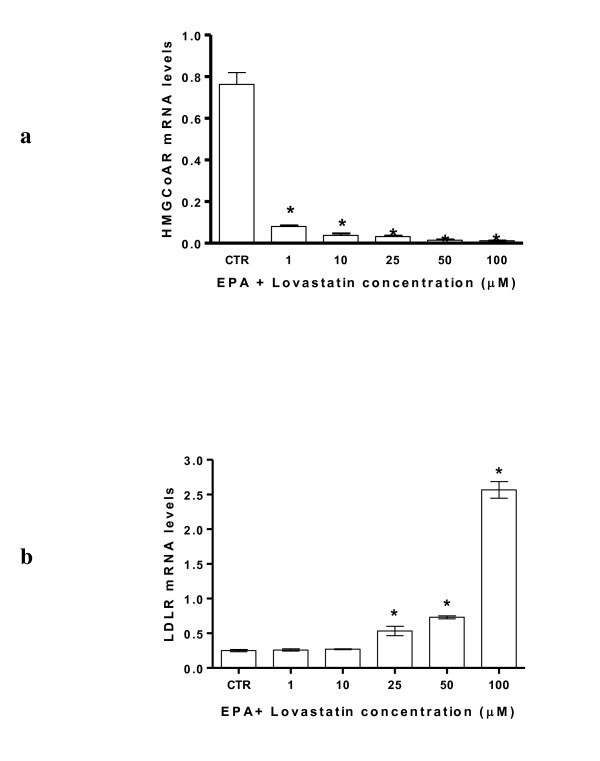
**HMGCoA reductase (panel a) and LDLR (panel b) mRNA levels after treatment with 50 μM of EPA and increasing concentrations of Lovastatin (1, 10, 25, 50 and 100 μM)**. All data represent the mean ± SE of four consecutive experiments. mRNA levels are expressed as ratio of the amount of gene target by the amount of β-actin. *P *value was determined by one way analysis with Dunnett' post test. *P < 0.05 versus control.

By the combination of both compounds, the effects on regulation of expression of both genes were detected at lower doses (Figure 3a and 3b).

**Figure 4:**
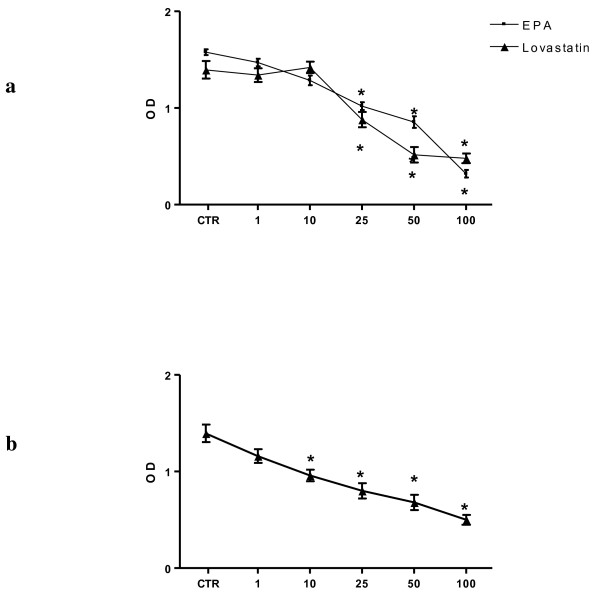
**Effect of increasing EPA and Lovastatin concentrations on the conversion of MTT-tetrazolium salt in HepG2 cells and the combined effect of EPA and Lovastatin on the conversion of MTT-tetrazolium salt in HepG2 cells**. *Panel a*: Effect of increasing EPA and Lovastatin concentrations on the conversion of MTT-tetrazolium salt in HepG2 cells. A significant anti-proliferative action was observed starting at 25 μM for both compounds used separately (*P < 0.05 Dunnett's multiple comparison test). *Panel b: *combined effect of EPA and Lovastatin on the conversion of MTT-tetrazolium salt in HepG2 cells (*P < 0.05 Dunnett's multiple comparison test).

In addition, EPA and Lovastatin, used separately, elicited a significant anti-proliferative effect in HepG2 cells starting at concentration of 25 μM (Figure 4a). The inhibition of cell proliferation was obtained at lower doses after combined treatment of EPA and Lovastatin (Figure 4b).

Cell growth inhibitory effect and gene regulation were similar using fixed EPA or Lovastatin, indifferently.

## Discussion

The anti-proliferative activity of EPA and statins has been suggested by numerous studies showing efficacy in smooth muscle, glioma, colon, ovarian and hepatoma cells [16,22-26].

In this study, we demonstrate the efficacy of EPA in regulation of lipid metabolism, as well as we confirm its anti-proliferative effects. Analysis of gene expression revealed in HepG2 cells an inhibition of HMGCoA reductase mRNA levels accompanied with an up-regulation of LDLR gene expression after EPA treatment. These effects, on both genes tested, were similar to those observed after Lovastatin exposure.

The combined treatment with EPA and lovastatin enhanced the regulatory effect on gene expression of HMGCoA reductase and LDL receptor in HepG2 cell line. Moreover, we detected a synergistic effect in the inhibition of cancer cell proliferation obtained by combination of EPA and Lovastatin.

The efficacy in regulation of gene expression and cell proliferation inhibition was detected at the lower doses with respect to the substances used separately. Furthermore, this synergistic effect of combined treatment was found to be not cytotoxic.

Previous studies have revealed that PUFA can regulate the expression of genes involved in several metabolic pathways [9,27]. Fish oil feeding, rich in the *n*-3 PUFA, drastically decreased mRNA levels for lipogenic enzymes in rodent liver [28]. PUFAs may control cholesterogenic gene expression through their effects on SREBP- dependent regulation [9]. SREBP-responsive genes include those coding for HMGCoA reductase and for intermediates in cholesterol synthesis, as Farnesyl Pirophosphate Synthase (FPPS). PUFAs decreased mRNA levels of lipogenic enzymes in rat hepatoma cells and mouse liver, in correlation with their effects on HMGCoA reductase [9].

The use of statins as monotherapy in the prevention or treatment of cancer has been limited in order to avoid potential detrimental effects that would have a negative impact on the health and well-being of the patient.

In order to avoid unwanted adverse side effects associated with high doses of statins, several studies have taken the approach to combine low doses of statins with other anticancer agents [29,30]. Experimental evidences suggest that statins and PUFAs have a reciprocal influence and the combined use of two compounds seems to have health beneficial effects [1,24].

The present work demonstrates that an additional advantage of synergistic effect could be the possible reduction of the doses of statin, thus limiting toxicity.

In conclusion, the pharmacological modulation of PUFAs combined with low doses of statins, even if merits further investigations in both preclinical and clinical settings, could be considered favorable in treatment of neoplastic diseases.

## Competing interests

The authors declare that they have no competing interests.

## Authors' contributions

MN and MGC conceived the study, participated in its design and coordination; CM, MGR, VT, and AM, performed various experiments; MN and MGC interpreted the data and wrote the manuscript; all authors read and approved the final manuscript.
